# Short-Term Effect of Micropulse Transscleral Laser Therapy on Intraocular Pressure in Untreated Fellow Eyes of Glaucoma Patients: Preliminary Results

**DOI:** 10.3390/jcm12113680

**Published:** 2023-05-26

**Authors:** Laura L. Fortuna, Thomas Dervos, Zisis Gatzioufas, Hendrik P. N. Scholl, Konstantin Gugleta, Tim J. Enz

**Affiliations:** 1 Department of Ophthalmology, University Hospital Basel, 4056 Basel, Switzerland; fortuna.laura@hotmail.com (L.L.F.); thomas.dervos@usb.ch (T.D.); zisis.gkatzioufas@usb.ch (Z.G.); hendrik.scholl@usb.ch (H.P.N.S.); konstantin.gugleta@usb.ch (K.G.); 2 Institute of Molecular and Clinical Ophthalmology Basel, 4056 Basel, Switzerland

**Keywords:** intraocular pressure, fellow eye, consensual ophthalmotonic reaction, micropulse transscleral laser treatment

## Abstract

It has been observed that an intraocular pressure (IOP) altering intervention in one eye is followed by a consensual response in the untreated fellow eye. The underlying mechanisms remain unclear. Involvement of neuronal, cytokine, and hormonal regulation of aqueous humor dynamics, as well as improved treatment adherence or systemic absorption of topically administered medical compounds, have been suggested. Our aim was to investigate the short-term effects of unilateral micropulse transscleral laser therapy on IOP in the fellow eye. All medical records of glaucoma patients who underwent micropulse transscleral laser therapy in a tertiary referral center between May 2019 and February 2023 were collected and analyzed. We found a significant reduction in IOP in the treated eyes, indicating successful treatment. In the fellow eyes, despite not having changed any of the pharmacological IOP-reducing therapies, a significant reduction in IOP from 17.0 ± 5.1 mmHg to 13.5 ± 4.4 mmHg (*p* < 0.01) was observed. This reduction was, however, short-term and reached statistical significance on the first postoperative day only. Our findings support the concept of consensual inter-eye responses to unilateral IOP changes. Further research is warranted to elucidate the mechanisms underlying this phenomenon.

## 1. Introduction

Glaucoma is among the most prevalent blinding disorders and is characterized by the progressive, irreversible degeneration of the retinal ganglion cells, which make up the optic nerve and project the electrophysiological signal of the visual circuit to the brain. Clinically, the disease manifests with deterioration of visual sensitivity and progressive visual field deficits [1]. Intraocular pressure (IOP) is determined by aqueous humor production and aqueous humor outflow, which are subject to autoregulatory mechanisms that maintain IOP within a physiological range. Elevated IOP, which is usually the result of reduced outflow capacity, is the most important glaucoma risk factor, and hence current treatment strategies aim to lower IOP, either pharmaceutically, by laser treatment, or surgically [2,3]. While surgical treatment options aim to increase outflow capacity, pharmacological and laser treatment may also target aqueous humor production [4,5,6].

Although of paramount clinical importance, the intrinsic, intraocular mechanisms that regulate IOP are still only poorly understood [7,8,9,10,11,12]. Moreover, systemic factors and even interactions with the fellow eye have been proposed to influence IOP. In 1924, Weekers first described IOP changes in untreated eyes following IOP lowering procedures in the contralateral eyes in an experimental study and termed this phenomenon a “consensual ophthalmotonic reaction” [13]. Although some of the reports are conflicting, a consensual IOP reduction in the untreated contralateral eye has later been shown in human subjects following pharmaceutical IOP-lowering treatment as well as selective laser trabeculoplasty or surgical trabeculectomy [14,15,16,17,18,19,20,21]. Notably, a consensual ophthalmotonic reaction may not only occur following an IOP decrease in one eye. Elevated IOP in the unaffected or untreated fellow eye has been observed after trauma or steroid response in the contralateral eye [22,23].

The mechanisms that guide consensual ophthalmotonic reactions are yet to be fully elucidated. Involvement of neuronal, hormonal, and cytokine regulation of aqueous flow dynamics, as well as systemic drug effects, have been suggested [20,24,25,26,27,28,29,30]. However, while a consensual contralateral IOP decrease after monocular pharmaceutical treatment may be explained by drug absorption and systemic effects, this cannot be the case in interventional procedures. Furthermore, previous studies examined inter-eye IOP changes only following interventional procedures that enhance outflow capacities, e.g., selective laser trabeculoplasty or trabeculectomy [17,20,21], yet not following procedures that reduce aqueous humor production, such as cyclophotocoagulation.

Micropulse transscleral laser treatment is a relatively novel cyclophotocoagulation technique using a probe that delivers short pulses of laser energy to the ciliary body, thereby inhibiting its aqueous humor production capacities [31,32]. Outflow capacities remain unchanged, and systemic pharmaceutical co-effects on the fellow eye can be excluded. The aim of this study is thus to analyze the effect of monocular micropulse transscleral laser therapy on IOP in the fellow eye in a cohort of glaucoma patients treated at our institution and hence to further shed light on the matter of consensual ophthalmotonic reactions and the mechanisms that may guide them.

## 2. Materials and Methods

### 2.1. Study Design and Ethics

This present analysis is a retrospective cohort study. The retrospective data analysis was conducted in accordance with the Declaration of Helsinki and was approved by the appropriate ethics committee (Ethikkommission Nordwest- und Zentralschweiz EKNZ). All participants gave written informed consent for their data to be analyzed.

### 2.2. Micropulse Transscleral Laser Therapy

**Figure 1:**
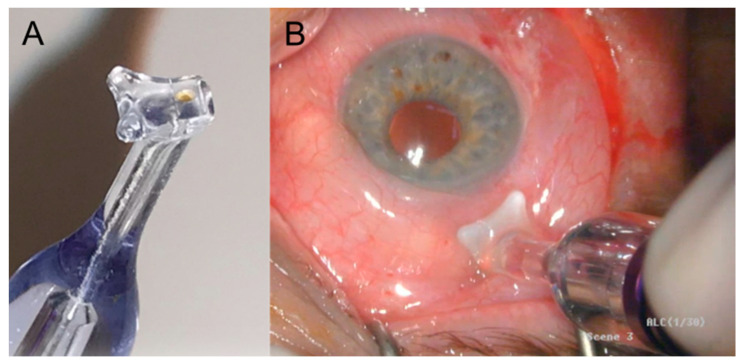
Photographs showing the MP3 laser probe for micropulse transscleral laser therapy (**A**) and a treatment procedure in progress (**B**).

All interventions were performed by the same surgeon (KG) in the operating theater. For pain management, topical anesthesia was applied using tetracaine drops and subconjunctival mepivacaine 1% injection, accompanied by systemic sedation. While the segments at 3 h and 9 h positions were spared, the superior and inferior perilimbal circumferences were treated under direct visual control using the MP3 probe (CycloG6 Laser, Iridex, Mountain View, CA, USA). Both the superior and the inferior hemicircles were treated for 90 s and with 3 Watts of energy at an application velocity of app. 20 s. Photographs of the MP3 laser probe, as well as a treatment procedure in progress, are shown in Figure 1.

### 2.3. Subjects and Retrospective Medical Records Reviewing

All patients who consecutively underwent micropulse transscleral laser treatment at the University Hospital Basel between May 2019 and February 2023 were identified using our clinical information software Medisight/Heyex EMR-1 (Heidelberg Engineering, Heidelberg, Germany), and those who fulfilled the inclusion criteria were included in the analysis. Inclusion criteria were the laser intervention on only one eye, concomitant yet better-controlled glaucoma or ocular hypertension on the contralateral eye, and the availability of intraocular pressure (IOP) readings at the same time point with the same method in both eyes preoperatively, 6–12 h after the intervention, on the first postoperative day, after one week and one month. Furthermore, patients whose pharmacological IOP-reducing therapy had been changed, be it in the operated or in the fellow eye, were excluded from the analysis. Next, patients’ medical records were scrutinized for patients’ age, sex, glaucoma type, IOP in both eyes before and following treatment, topical and systemic IOP lowering medication, as well as intra- and postoperative complications.

### 2.4. Statistical Analysis

Differences in preinterventional IOP and IOP at 6–12 h after the intervention, on the first postinterventional day, after one week and one month, in the treated eyes as well as the fellow eyes, as independent samples, were defined as primary outcomes. All statistical analyses were performed in Statistica, Version 13.5.0.17 (TIBCO Software Inc., Palo Alto, CA, USA) program. Descriptive analysis with calculation of mean values, standard deviation, median values, and ranges was performed. Data were tested for normality with a Shapiro–Wilk and Kolmogorov–Smirnov test, and since they were normally distributed, they were compared by a non-paired and paired Student’s *t*-test. Correlation analysis was performed, with correction for multiple comparisons. *p* < 0.05 was considered statistically significant.

## 3. Results

A total of 22 patients (*n* = 22) were included in this study. In 12 patients, the right eyes were operated on, while in 10 patients, the left eyes were operated on. The patients’ mean age was 74.5 years. A total of 13 patients were female. Most of the treated and fellow eyes were primary open-angle glaucomas (15/15), and the rest were secondary open-angle glaucomas (pseudoexfoliation and pigment dispersion glaucoma). In the fellow eye group, there were four eyes without any topical IOP-reducing therapy. The average number of running IOP-reducing medications in the interventional eyes was 2.6 (range 2–5), and in the fellow eyes, 1.7 (range 0–4), where each compound of each substance group was scored as 1 (beta-blockers, alpha-2-agonists, carbonic-anhydrase inhibitors—scored separately for topical and systemic application—and prostaglandin analogs).

Student’s *t*-test for dependent samples revealed significant IOP reduction compared to baseline at all time points in the treated eyes. In the fellow eyes, a significant IOP reduction compared to baseline was found on the first postoperative day. At one week and at one month postoperatively, IOP in the untreated fellow eyes was still lower compared to baseline, but this was not statistically significant. There was no significant correlation in individual pressure changes in the two eyes at any of the parallel IOP readings. Mean IOP values in the interventional and the fellow eyes prior to intervention, postoperatively on the same day, on the first postoperative day, one week later, and one month later, are depicted in Table 1, together with the native *p*-value of the paired *t*-test with the corresponding baseline (preoperative) IOP level.

No relevant intra- or postoperative complications concluding one month after the intervention were observed, apart from low-grade irritation of the anterior chamber in laser-treated eyes and temporary discrete visual acuity reduction.

## 4. Discussion

Although the underlying systemic mechanisms may be of paramount scientific and clinical relevance as a possible treatment target, the phenomenon of IOP changes in fellow eyes following unilateral IOP-changing treatment of the contralateral eye is only poorly understood, and published evidence on this topic is controversial. In 1924, Weekers was the first to describe “consensual ophthalmotonic reactions” based on the observations of his experimental study [13]. In 1927, Wilmer reported a consensual binocular decrease in IOP in rabbit eyes following monocular fistulating surgery, supporting Weekers’ observations [33]. Another animal study found a rise in IOP in the fellow eye after paracentesis in one eye, suggesting that consensual IOP change can also be in the opposite direction [34]. Later on, IOP reductions in untreated fellow eyes were reported in humans unilaterally exposed to IOP-lowering eye drops, ocular compression, or laser trabeculoplasty [14,15,16,17,18,19,21], followed by reports of (temporary) consensual contralateral IOP decreases after unilateral trabeculectomy in glaucoma patients [30,35]. On the other hand, a post hoc analysis of data from the Collaborative Initial Glaucoma Treatment Study revealed no evidence of a substantial effect of trabeculectomy on the IOP in the fellow eyes [36], and some studies even found an increase in IOP in the fellow eye following trabeculectomy or aqueous drainage device implantation in the contralateral eye [29,37,38].

The inconsistency and conflicting observations in published evidence may be due to differences in study designs, inclusion and exclusion criteria, surgical techniques, or observational follow-up periods. In many of the above-mentioned studies, a number of limitations were identified, e.g., small sample sizes, different surgeons within the same studies, IOP measurements by different types of tonometers, etc. Furthermore, in many studies, conditions in the untreated eyes that may have influenced IOP during the observation period were not sufficiently elaborated (previous or existing IOP-lowering therapy, narrow angle, or pseudoexfoliation). Nonetheless, evidence supporting the existence of a consensual ophthalmotonic reaction appears to prevail.

The mechanisms that could guide such inter-eye responses are unclear, yet a number of different theories have been proposed. In 1924, shortly after Weekers first described his observations of consensual ophthalmotonic reactions, Leplat demonstrated that blunt trauma to rabbit eyes produced a more pronounced consensual IOP rise in the fellow eye if the animal was not anesthetized, suggesting that central regulation mechanisms are involved. Various experimental studies found evidence for hypothalamus, sympathetic and parasympathetic activity during possible consensual ophthalmotonic reactions as a sign of neuronal regulation [22,25,26,27,28,39]. Al-Ghadyan et al., on the other hand, suggested a trauma-related increase in prostaglandin release as a possible explanation for the contralateral IOP-spike after monocular paracentesis in rabbits found in his study [34]. Kaushik et al. mentioned the possible involvement of cytokine and hormonal activities regulating aqueous humor dynamics as explanations for consensual IOP changes in fellow eyes [38]. Yet, other scholars attribute the consensual behavior in IOP fluctuations to confounding factors such as systemic absorption of IOP-lowering eye drops, stricter application of IOP-lowering eye drops during the hospital stay, a temporarily lasting effect of perorally taken acetacolamide before surgery, or improved patient compliance in medication following surgical intervention. Furthermore, it has been postulated that glaucoma surgery is usually scheduled when there is a peak in IOP, and hence the IOP decrease in the untreated fellow eye might be the result of a “regression to the mean” effect [20,38,40]. Synoptically, the matter of consensual ophthalmotonic reactions remains elusive.

In our study, we retrospectively assessed changes in IOP before and following unilateral micropulse transscleral laser therapy and found a significant IOP reduction in the untreated fellow eyes on the first postoperative day. Since topical and/or systemic IOP-lowering therapy remained unchanged, an effect attributed to more effective pharmacological treatment (e.g., perorally administered acetacolamide) or systemic absorption of IOP-lowering eye drops can be excluded. While previous studies assessed inter-eye IOP responses following surgical interventions that increase aqueous humor outflow, this is, to the very best of our knowledge, the first study to investigate changes in untreated fellow eyes following a procedure that non-pharmacologically decreases aqueous humor production in the contralateral eyes. Hence, an effect mediated by changes in flow through the trabecular meshwork of the treated eyes appears unplausible as well. Although the stricter application of IOP-lowering eye drops during the hospital stay cannot be fully excluded, the results of our study appear to support the concept of central or systemic IOP co-regulation and mechanisms that mutually affect IOP in both eyes.

Our study is limited by the small sample size, the retrospective design, the single-center observational nature, and the lack of controls, and hence our findings may be considered preliminary. Moreover, our study does not provide any additional information on the possible nature of the presumed systemic mechanisms that regulate IOP, as well as possible IOP-related interactions between the eyes. Prospective and more sophisticated studies are necessitated to further elucidate these mechanisms. A better comprehension of these processes may improve our understanding of IOP dynamics in health and disease and ultimately lead to enhanced treatment options in elevated IOP and glaucoma. Furthermore, a consensual decrease in IOP in untreated fellow eyes that is more sustained than was found in our study might also be considered an option for at least temporary treatment of elevated IOP in the eye that is not (yet) to be operated on, and hence, might allow additional time and facilitate treatment scheduling.

## 5. Conclusions

Micropulse transcleral laser therapy in one eye of glaucoma patients resulted in a significant reduction in IOP in the treated eye, indicating successful treatment. In the fellow eye, despite not having changed the pharmacological IOP-reducing therapy, be it systemically or topically, a significant reduction in IOP was observed. This reduction was, however, short-term and reached statistical significance on the first postoperative day only. Our findings support the concept of consensual inter-eye responses to unilateral IOP changes. Further research is warranted to elucidate this phenomenon.

## Figures and Tables

**Table 1:** Mean intraocular pressure values and standard deviation in interventional and untreated fellow eyes as measured preoperatively as well as postoperatively on the same day of the intervention, 1 day, 1 week, and 1 month later. Native *p*-values of the paired *t*-test with the corresponding preoperative IOP level.

Visit	Interventional Eyes	*p* Value	Fellow Eyes	*p* Value
Preintervention	31.5 ± 14.1 mmHg		17.0 ± 5.1 mmHg	
Same-day postintervention	30.1 ± 11.2 mmHg	0.697	15.8 ± 5.6 mmHg	0.221
1-day postintervention	22.5 ± 10.6 mmHg	0.003	13.5 ± 4.4 mmHg	0.001
1-week postintervention	16.4 ± 6.8 mmHg	<0.001	15.1 ± 3.8 mmHg	0.066
1-month postintervention	19.4 ± 8.8 mmHg	<0.001	16.0 ± 4.3 mmHg	0.308

## Data Availability

All data analyzed in this study are available from the corresponding author upon reasonable request.

## References

[1] Jonas J.B., Aung T., Bourne R.R., Bron A.M., Ritch R., Panda-Jonas S. (2017). Glaucoma. Lancet.

[2] Tan J.C., Peters D.M., Kaufman P.L. (2006). Recent developments in understanding the pathophysiology of elevated intraocular pressure. Curr. Opin. Ophthalmol..

[3] Conlon R., Saheb H., Ahmed I.I. (2017). Glaucoma treatment trends: A review. Can. J. Ophthalmol..

[4] Kang J.M., Tanna A.P. (2021). Glaucoma. Med. Clin. N. Am..

[5] Lim R. (2022). The surgical management of glaucoma: A review. Clin. Exp. Ophthalmol..

[6] Casson R.J. (2022). Medical therapy for glaucoma: A review. Clin. Exp. Ophthalmol..

[7] Johnstone M.A. (2004). The aqueous outflow system as a mechanical pump: Evidence from examination of tissue and aqueous movement in human and non-human primates. J. Glaucoma.

[8] Johnstone M., Martin E., Jamil A. (2011). Pulsatile flow into the aqueous veins: Manifestations in normal and glaucomatous eyes. Exp. Eye Res..

[9] Gonzalez J.M., Ko M.K., Hong Y.K., Weigert R., Tan J.C.H. (2017). Deep tissue analysis of distal aqueous drainage structures and contractile features. Sci. Rep..

[10] Johnstone M.A. (2014). Intraocular pressure regulation: Findings of pulse-dependent trabecular meshwork motion lead to unifying concepts of intraocular pressure homeostasis. J. Ocul. Pharmacol. Ther..

[11] Carreon T., van der Merwe E., Fellman R.L., Johnstone M., Bhattacharya S.K. (2017). Aqueous outflow—A continuum from trabecular meshwork to episcleral veins. Prog. Retin. Eye Res..

[12] Lusthaus J.A., Khatib T.Z., Meyer P.A.R., McCluskey P., Martin K.R. (2021). Aqueous outflow imaging techniques and what they tell us about intraocular pressure regulation. Eye.

[13] Weekers L. (1924). Modification expérimentales de l’ophtalmotonous. Reaction ophtalmotonique consensuelle. Arch. Opthalmol..

[14] Piltz J., Gross R., Shin D.H., Beiser J.A., Dorr D.A., Kass M.A., Gordon M.O., The Ocular Hypertension Treatment Study Group (2000). Contralateral effect of topical beta-adrenergic antagonists in initial one-eyed trials in the ocular hypertension treatment study. Am. J. Ophthalmol..

[15] Kwitko G.M., Shin D.H., Ahn B.H., Hong Y.J. (1987). Bilateral effects of long-term monocular timolol therapy. Am. J. Ophthalmol..

[16] Martin X.D., Rabineau P.A. (1988). Intraocular pressure effects of timolol after unilateral instillation. Ophthalmology.

[17] Rhodes K.M., Weinstein R., Saltzmann R.M., Aggarwal N., Kooner K.S., Petroll W.M., Whitson J.T. (2009). Intraocular pressure reduction in the untreated fellow eye after selective laser trabeculoplasty. Curr. Med. Res. Opin..

[18] Gibbens M.V. (1988). The consensual ophthalmotonic reaction. Br. J. Ophthalmol..

[19] Newman H., Kurtz S., David R. (2010). Intraocular pressure changes in the contralateral eye after topical treatment: Does an “ophthalmotonic consensual reaction” exist?. Isr. Med. Assoc. J..

[20] Aghayeva F.A., Chronopoulos P., Schuster A.K., Pfeiffer N., Hoffmann E.M. (2021). Inter-eye relationship of intraocular pressure change after unilateral trabeculectomy, filtering canaloplasty, or PreserFlo™ microshunt implantation. Graefes Arch. Clin. Exp. Ophthalmol..

[21] Liu Y., Fan X., Wu L. (2022). Selective laser trabeculoplasty lowered the untreated fellow eye long-term intraocular pressure: A 3-year observational study. Lasers Med. Sci..

[22] Leplat G. (1924). Etude de quelques reactions dans les yeux par une contusion oculaire unilateral; recherches experimentales et cliniques. Ann. Oculist.

[23] Pandit R., George R.J., Lingam V., Balekudaru S. (2021). Incidence of presumed steroid response in contralateral eye of patients who underwent glaucoma filtration surgery. Indian. J. Ophthalmol..

[24] Diestelhorst M., Krieglstein G. (1991). The effect of trabeculectomy on the aqueous humor flow of the unoperated fellow eye. Graefes Arch. Clin. Exp. Ophthalmol..

[25] Schmerl E., Steinberg B. (1948). Central control of intraocular pressure by active principles. Am. J. Ophthalmol..

[26] Gloster J., Greaves D.P. (1957). Effect of diencephalic stimulation upon intra-ocular pressure. Br. J. Ophthalmol..

[27] Cox C.E., Fitzgerald C.R., King R.L. (1975). A preliminary report on the supraoptic nucleus and control of intraocular pressure. Invest. Ophthalmol..

[28] Gibbens M.V. (1988). Sympathetic influences on the consensual ophthalmotonic reaction. Br. J. Ophthalmol..

[29] Yarangümeli A., Köz O.G., Kural G. (2003). The effect of trabeculectomy on the intraocular pressure of the unoperated fellow eye. J. Glaucoma.

[30] Vysniauskiene I., Shaarawy T., Flammer J., Haefliger I.O. (2005). Intraocular pressure changes in the contralateral eye after trabeculectomy with mitomycin, C. Br. J. Ophthalmol..

[31] Abdelmassih Y., Tomey K., Khoueir Z. (2021). Micropulse Transscleral Cyclophotocoagulation. J. Curr. Glaucoma Pract..

[32] Sanchez F.G., Peirano-Bonomi J.C., Brossard Barbosa N., Khoueir Z., Grippo T.M. (2020). Update on Micropulse Transscleral Cyclophotocoagulation. J. Glaucoma.

[33] Wilmer W.E. (1927). Discussion on the results of operative treatment of glaucoma. Trans. Ophthalmol. Soc..

[34] Al-Ghadyan A., Mead A., Sears M. (1979). Increased pressure after paracentesis of the rabbit eye is completely accounted for by prostaglandin synthesis and release plus pupillary block. Invest. Ophthalmol. Vis. Sci..

[35] Detorakis E.T., Tsiklis N., Pallikaris I.G., Tsilimbaris M.K. (2011). Changes in the intraocular pressure of fellow untreated eyes following uncomplicated trabeculectomy. Ophthalmic Surg. Lasers Imaging.

[36] Radcliffe N.M., Musch D.C., Niziol L.M., Liebmann J.M., Ritch R., CIGTS Study Group (2010). The effect of trabeculectomy on intraocular pressure of the untreated fellow eye in the collaborative initial glaucoma treatment study. Ophthalmology.

[37] Shum J.W.H., Choy B.N.K., Ho W.L., Chan J.C.H., Lai J.S.M. (2016). Consensual ophthalmotonic reaction in Chinese patients following augmented trabeculectomy or ExPRESS shunt implantation. Medicine.

[38] Kaushik S., Agarwal A., Kaur S., Lomi N., Raj S., Pandav S.S. (2016). Change in Intraocular Pressure in the Fellow Eye After Glaucoma Surgery in 1 Eye. J. Glaucoma.

[39] Rajurkar K., Pegu J., Dubey S., Kumar A., Singh N. (2018). Analysis of Fellow Eye Intraocular Pressure Changes after Glaucoma Surgery in 1 Eye. J. Glaucoma.

[40] Leung D.Y., Kwong Y.Y., Yuen H.K. (2006). Intraocular pressure changes in the contralateral eye after trabeculectomy with mitomycin, C. Br. J. Ophthalmol..

